# Prescription Digital Therapeutics Research Across Clinical, Engagement, Regulatory, and Implementation Domains: A Bibliometric and Thematic Study

**DOI:** 10.7759/cureus.84528

**Published:** 2025-05-21

**Authors:** Shaheen E Lakhan

**Affiliations:** 1 Medical Office, Click Therapeutics, Inc., New York, USA; 2 Department of Neurology, Western University of Health Sciences, Pomona, USA; 3 Department of Neurology, A.T. Still University School of Osteopathic Medicine in Arizona, Mesa, USA; 4 Department of Neurology, Morehouse School of Medicine, Atlanta, USA; 5 Department of Bioscience, Global Neuroscience Initiative Foundation, Miami, USA

**Keywords:** bibliometric analysis, clinical science, digital health, engagement science, implementation science, pdt, prescription digital therapeutics, regulatory science, software as a medical device, topic modeling 4o

## Abstract

Prescription digital therapeutics (PDTs) are Food and Drug Administration (FDA)-authorized software-based treatments designed to treat a range of conditions on the smartphone. Their development and deployment rely on four foundational scientific domains: clinical, engagement, regulatory, and implementation. However, the relative representation of these domains in the PDT literature has not been systematically characterized.

We conducted a bibliometric and thematic analysis of PubMed-indexed articles published between 2020 and 2025 containing the term “prescription digital therapeutic(s).” Metadata and abstracts were extracted, cleaned, and analyzed using natural language processing for this review. Topic modeling was performed to identify key thematic areas, and each abstract was classified into one or more of the four foundational domains using a structured keyword heuristic framework. Trends in publication volume, authorship, domain co-occurrence, and thematic focus were visualized.

Sixty-one unique articles met the inclusion criteria. Publication activity increased over time, peaking in 2022 and 2024. Most first authors were based in the United States, with industry-affiliated authorships predominating. The most frequently publishing journals were Frontiers in Psychiatry and Health Affairs (Millwood). Clinical science was referenced in 45 (74%) papers, followed by engagement science in 35 (58%), regulatory science in 28 (46%), and implementation science in 18 (29%). Only seven (12%) articles addressed all four domains. Topic modeling identified five major themes: substance use and cost modeling, regulatory frameworks, insomnia treatment, engagement strategies, and gamified pediatric interventions. Co-occurrence analysis revealed strong overlap between clinical and engagement domains, while regulatory and implementation science appeared less frequently in combination.

The literature on PDTs remains concentrated in clinical and engagement domains, with limited attention to regulatory strategy and real-world implementation. Greater integration across all four scientific domains is needed to ensure that PDTs are not only effective but also scalable, fundable, and embedded into routine care.

## Introduction and background

Prescription digital therapeutics (PDTs) represent a transformative class of medical treatments built entirely on software. These interventions are not mere wellness tools; rather, they are rigorously evaluated, often Food and Drug Administration (FDA)-cleared, and designed to deliver clinical benefit for specific medical indications [1]. As of 2025, PDTs have been cleared for a diverse range of conditions, including substance use disorders [2], major depressive disorder [3], insomnia [4], type 2 diabetes [5], and migraine [6]. These advancements reflect a convergence of evidence-based medicine, regulatory innovation, and digital design.

Unlike traditional therapeutics that are built upon a foundation of pharmacokinetics and pharmacodynamics, PDTs draw from multiple scientific disciplines. To systematically study and support their development, it is necessary to articulate a framework that categorizes the types of science that underlie their conception, validation, and integration. PDTs are grounded in four foundational sciences: clinical science, engagement science, regulatory science, and implementation science [1,7]. Clinical science focuses on generating evidence through methodologies such as randomized controlled trials (RCTs), observational studies, and validated endpoints. Engagement science investigates how digital interventions sustain user participation, adherence, and behavior change. Regulatory science explores the design and application of evaluation frameworks for software as a medical device (SaMD), including pathways such as 510(k) and De Novo. These regulatory structures continue to evolve in response to the unique characteristics of software-based interventions [8]. Implementation science ensures that these innovations move beyond controlled environments into real-world settings, accounting for workflows, payer coverage, equity, and scalability.

While these domains are often referenced in PDT literature, their relative representation in published research remains poorly understood. Most studies focus on clinical efficacy, but it is unclear how often engagement, regulatory, or implementation concerns are explicitly addressed. The lack of visibility into these foundational components presents a gap for strategic research investment, regulatory alignment, and commercial readiness.

The objective of this review is to evaluate the representation of clinical, engagement, regulatory, and implementation sciences in the published literature on PDTs. By applying bibliometric and thematic analysis methods to PubMed-indexed articles, we aim to identify dominant trends, topic areas, and gaps across these foundational domains. By doing so, we aim to clarify which areas are well-represented in the literature and which remain underexplored, offering guidance for future scholarship and development.

## Review

Data sources and selection criteria

A structured search of PubMed was conducted using the terms “prescription digital therapeutic” and “prescription digital therapeutics.” The search spanned from the earliest available to May 4, 2025. Articles were included if they discussed PDTs, clinical trials, real-world evidence, regulatory submissions, or implementation. Metadata including PubMed ID (PMID), title, authors, journal, year, digital object identifier (DOI), and author affiliations were exported in comma-separated values (CSV) format, while full abstracts were collected in a separate text file. Country of first author affiliation and institutional setting (academic, clinical, industry, regulatory, and others) were manually extracted from PubMed records to supplement bibliometric analysis. Abstracts were cleaned and matched to metadata using custom scripts.

Bibliographic parsing and cleaning

Using heuristic rules and regular expression-based parsing, we extracted the longest informative paragraph in each entry as the presumed abstract. Copyright lines, conflict of interest disclosures, and redundant identifiers were excluded. The dataset was manually reviewed to ensure alignment between titles, abstracts, and PMIDs.

Topic modeling

To identify latent themes, we applied nonnegative matrix factorization (NMF) to a term frequency-inverse document frequency (TF-IDF) matrix generated from the abstract corpus [9]. We selected NMF for topic modeling based on its strong interpretability in sparse textual datasets. Further, we identified five components based on coherence and interpretability. Top keywords from each topic were extracted and used to assign preliminary themes.

Domain classification framework

Each abstract was manually or semiautomatically classified into one or more of the following foundational scientific domains based on keyword heuristics (Table 1) [10]. Clinical science pertains to the generation of evidence for safety, efficacy, and outcomes using methods such as clinical trials and validated symptom scales. Engagement science encompasses behavior change strategies, patient adherence, therapeutic alliance, and interaction design. Regulatory science involves FDA clearance, SaMD classification, labeling, and regulatory pathways, including De Novo and 510(k). Implementation science addresses the adoption of PDTs into routine care, health system workflows, payer coverage, and considerations of health equity. The domain classification framework was developed to organize and quantify the representation of core scientific disciplines supporting PDT development. This model allowed systematic tagging of each article by foundational domain to reveal trends and gaps. Keyword matching was implemented in Python (v3.13.3; Python Software Foundation, Wilmington, DE). Each abstract was tagged with one or more domains based on keyword presence. Ambiguous or borderline cases were resolved through manual review.

**Table 1:** Foundational Domain Classification Framework This author-created table defines the four core scientific domains used to classify prescription digital therapeutics (PDT) literature. Each domain is accompanied by a brief definition and representative keywords used for heuristic classification of papers. Regulatory pathways such as 510(k) and De Novo are part of the US Food and Drug Administration (FDA) clearance process for software as a medical device (SaMD). The framework was applied to identify the primary scientific focus of each article in the analysis RCT: randomized controlled trial

Domain	Definition	Example keywords
Clinical science	Evidence generation for safety, efficacy, and outcomes	efficacy, RCT, endpoint, symptom, and clinical trial
Engagement science	Behavior change, adherence, motivation, and therapeutic alliance	adherence, engagement, gamification, retention, and behavior change
Regulatory science	Pathways for clearance, labeling, validation, and post-market surveillance	FDA, De Novo, 510(k), SaMD, labeling, and regulatory
Implementation science	Adoption into care, health system integration, payer coverage, scalability, and equity	real-world, implementation, equity, payer, and integration

Visualization and analysis

We used matplotlib and seaborn (NumFOCUS, Austin, TX) for visualizations [11]. A binary matrix was constructed to indicate the presence or absence of each of the four foundational domains in each article. From this, a domain co-occurrence matrix was derived, where diagonal values represent total article-level assignments per domain, and off-diagonal cells reflect the number of articles assigned to both domains. The matrix was visualized as a heatmap, with shading intensity corresponding to the frequency of co-occurrence.

Bibliometric overview

A total of 61 unique articles met the inclusion criteria. Publication activity began in 2020 and increased over the five-year period, with the greatest number of articles appearing in 2022 and 2024. Each of those years saw 15 publications, representing nearly half of all articles in the dataset. This trend corresponds with significant milestones in PDT regulatory approvals, expanded commercial investment, and broadening academic interest. Fewer articles were published in 2020 and 2021, reflecting the nascent stage of PDT scholarship during those years, while 2025 shows a partial count through early May.

The most frequently publishing journals were Frontiers in Psychiatry and Health Affairs (Millwood), followed by Advances in Therapy and Current Medical Research and Opinion (Table 2). First authors were based primarily in the United States, with occasional contributions from India, Canada, Australia, and the United Kingdom (Table 3). The majority of articles included authors affiliated with industry, while a smaller proportion was led by academic, clinical, or regulatory institutions.

**Table 2:** Top 10 Journals Publishing PDT Literature This author-created table lists the 10 journals with the highest number of PubMed-indexed articles on prescription digital therapeutics (PDTs) published between 2020 and 2025. Journal names are ordered by article count, with ties resolved based on the earliest year of publication JMIR: Journal of Medical Internet Research

Journal	Article Count
Frontiers in Psychiatry	5
Health Affairs (Millwood)	5
Advances in Therapy	4
Current Medical Research and Opinion	3
Expert Review of Pharmacoeconomics & Outcomes Research	3
Frontiers in Digital Health	3
Hospital Practice	2
Journal of Managed Care & Specialty Pharmacy	2
JMIR Formative Research	2
JMIR Research Protocols	2

**Table 3:** Country of First Author Affiliation This author-created table summarizes the countries of first author affiliation for the 61 included articles on prescription digital therapeutics (PDTs). Article counts reflect the number of publications per country between 2020 and 2025, with ties ordered by the earliest year of appearance in the dataset

Country	Article Count
United States	57
India	1
Canada	1
Australia	1
United Kingdom	1

Topic modeling findings

The NMF model identified five major themes (Table 4). One topic clustered around substance use disorders and cost modeling, with keywords such as reSET, opioid use disorder (OUD), medication-assisted therapy (MAT), and cost-effectiveness, representing literature on addiction-related PDTs and health economic analyses. A second topic highlighted digital health regulation and industry trends, with prominent terms such as SaMD, FDA, 510(k), and clearance, reflecting the intersection of product development and regulatory navigation. A third topic centered on insomnia and sleep treatments, with Somryst, cognitive-behavioral therapy for insomnia(CBT-I), insomnia severity index (ISI), and sleep efficacy frequently noted, reflecting a coherent body of literature on digital CBT-I. The fourth topic focused on engagement and behavior change, identifying keywords such as adherence, motivation, retention, behavior, and user alliance. The fifth and final cluster captured gamification and pediatric attention-deficit/hyperactivity disorder (ADHD) interventions, especially those using gameplay mechanics, with keywords such as EndeavorRx, attention, children, and gamified design. These themes were consistent with key PDT indications and business drivers. Engagement-oriented language was observed across multiple clusters, reinforcing its central role even in studies with a clinical or regulatory focus.

**Table 4:** Thematic Topics Identified From Bibliographic Analysis This author-created table presents five major themes identified through topic modeling of bibliographic data on prescription digital therapeutics (PDTs). Each theme is labeled according to its content focus and includes top keywords that contributed to its characterization. Keywords include references to the US Food and Drug Administration (FDA), software as a medical device (SaMD), 510(k) premarket notification pathway, cognitive-behavioral therapy for insomnia (CBT-I), insomnia severity index (ISI), medication-assisted therapy (MAT), and attention-deficit/hyperactivity disorder (ADHD). The topics reflect prominent areas of scientific and clinical emphasis in PDT-related publications from 2020 to 2025 OUD: opioid use disorder

Topic	Theme	Top Keywords
1	Substance use and cost modeling	reSET, OUD, MAT, cost, abstinence, treatment, and economics
2	Regulatory and industry trends	SaMD, regulatory, FDA, 510(k), pathway, and clearance
3	Insomnia and sleep treatments	Somryst, CBT-I, insomnia, ISI, sleep, and efficacy
4	Engagement and behavior change	adherence, motivation, retention, behavior, user, and alliance
5	Gamification and pediatric ADHD	EndeavorRx, ADHD, focus, attention, children, and gamified

Domain representation

Clinical science was the most commonly represented domain, present in 45 (74%) papers. Engagement science appeared in 35 (58%), regulatory science in 28 (46%), and implementation science in 18 (29%). Only seven (12%) papers covered all four domains. In total, these classifications resulted in 292 article-level domain assignments across the 61 articles, reflecting that most studies addressed multiple domains. Most papers, 32 (52%), engaged with two domains, while 18 (30%) addressed three. A minority of four (6%) represented just one domain.

Domain co-occurrence

**Figure 1:**
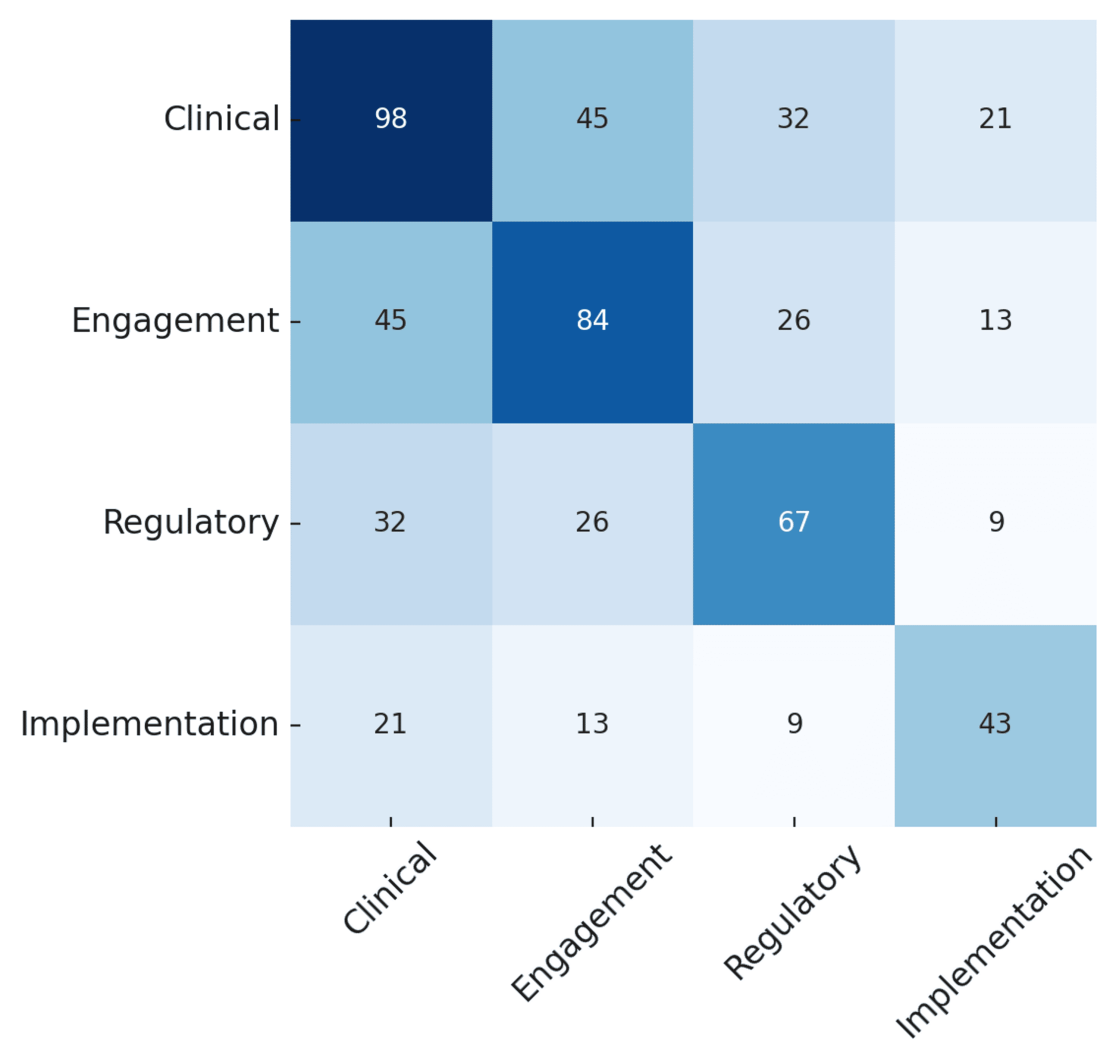
Domain Co-occurrence Heatmap This author-generated heatmap illustrates how often foundational scientific domains co-occurred within the same article. Diagonal values represent the number of article-level domain assignments for each individual domain. Off-diagonal values reflect the number of unique articles in which both domains were assigned. Because articles may be tagged with multiple domains, the total number of counts in the matrix exceeds the number of unique articles (n = 61). Shading intensity corresponds to frequency, with darker shades indicating more frequent assignment or co-occurrence

A domain co-occurrence heatmap (Figure 1) revealed strong alignment between clinical and engagement science, which co-occurred in 45 unique articles. Off-diagonal cells in the matrix represent how many articles were tagged with both domains, while diagonal cells reflect how many article-level domain assignments were made to each domain individually. For example, clinical science was assigned in 98/292 (33.6%) instances across the dataset, engagement in 84/292 (28.8%), regulatory in 67/292 (22.9%), and implementation in 43/292 (14.7%). Because most articles were assigned to multiple domains, total counts in the matrix exceed the number of unique articles. The most frequent pairings involved clinical science, especially with engagement, regulatory, or implementation. In contrast, regulatory and implementation science co-occurred in only nine articles, highlighting a gap between product clearance and real-world integration.

Domain overlap distribution

The majority of papers addressed multiple domains. Specifically, seven (12%) covered all four foundational domains, 18 (30%) addressed three domains, 32 (52%) addressed two, and only four (6%) were confined to a single domain. This distribution suggests that while interdisciplinary integration is occurring, few studies comprehensively incorporate all four foundational sciences.

Discussion

This study reveals an asymmetrical landscape in the published literature on PDTs. Clinical validation remains the dominant focus, likely reflecting the emphasis on evidence-based approval pathways and the necessity of demonstrating benefit through trials. Engagement science also appears frequently but is often addressed in superficial terms such as user adherence or retention, without reference to deeper theoretical models such as the capability, opportunity, motivation, behavior (COM-B) framework [12]; self-determination theory [13]; or therapeutic alliance [14]. Regulatory science is moderately represented and typically limited to describing FDA clearance, labeling, or SaMD categorization. However, there is minimal discussion of how regulatory precedents shape product design, labeling, usability, and trial endpoints, despite FDA guidance that increasingly accounts for the distinct lifecycle and risks of digital therapeutics [8]. Implementation science remains the least represented domain, despite its central role in enabling reimbursement, provider uptake, and integration into clinical workflows. To accelerate progress, future PDT studies should embed engagement and implementation strategies early in the design process and report on real-world uptake outcomes. Funding agencies and journal editors could also help rebalance the field by prioritizing research that goes beyond clinical efficacy to include usability, equity, and health system readiness.

Future research should more thoroughly incorporate engagement and implementation perspectives from the earliest stages of product development. For researchers, this may involve including digital behavior change frameworks and implementation outcome measures in study design. For developers, it means ensuring that regulatory rationale, usability testing, and workflow compatibility are documented as thoroughly as clinical endpoints. For payers and regulators, establishing expectations for implementation reporting and equity assessments could foster broader uptake and transparency.

The gaps identified also point to opportunities for interdisciplinary collaboration. As the digital therapeutics field grows, studies that bring together regulatory experts, behavioral scientists, implementation researchers, and clinical trialists are likely to be more impactful and aligned with real-world needs. Bridging these domains is essential to fulfilling the promise of PDTs.

This analysis also has implications for the digital therapeutics industry. A more balanced foundation of supporting sciences can enhance credibility, payer trust, and patient benefit. Addressing implementation gaps, for example, could facilitate reimbursement decisions and support integration into formularies. Similarly, articulating regulatory precedents and aligning on labeling conventions could accelerate market entry and reduce uncertainty.

Limitations

This study has several limitations. First, it relies on PubMed-indexed abstracts, which may not capture the full scope of methodological detail, especially regarding engagement, regulatory, and implementation considerations that may appear only in the full text. Second, our keyword-based classification framework, while systematic and informed by prior domain definitions, may undercount or overcount domain representation due to variation in terminology or phrasing. Third, the topic modeling approach reflects thematic clusters based on word co-occurrence patterns and does not guarantee conceptual exclusivity or completeness. Fourth, author institutional affiliations were classified manually based on PubMed listings, which may not always clearly reflect primary professional roles or dual affiliations. Fifth, the dataset is restricted to English-language, PubMed-indexed publications. Although this may bias general bibliometric studies toward high-income country research, the risk is mitigated in this review due to its focus on US FDA-cleared PDTs, which are primarily developed, trialed, and published within PubMed-indexed literature. Last, this study followed bibliometric review conventions rather than systematic review guidelines such as Preferred Reporting Items for Systematic Reviews and Meta-Analyses (PRISMA), which are more appropriate for meta-analyses of clinical trials. These limitations may bias the representation of foundational scientific domains in the PDT literature and should be addressed in future research using more comprehensive datasets and full-text analysis.

## Conclusions

This bibliographic analysis and review provide the first structured analysis of how foundational scientific domains (clinical, engagement, regulatory, and implementation) are represented in the published literature on FDA-cleared PDTs. Clinical and engagement sciences dominate, while regulatory and implementation aspects remain underexplored, despite their essential roles in bringing PDTs to market and into clinical practice.

A major strength of this study is the application of a structured domain framework and topic modeling to classify and interpret the literature, offering a replicable method for future analyses. Limitations include reliance on PubMed abstracts, potential keyword bias, and the exclusion of non-indexed or grey literature. However, given the US regulatory focus of this field, relevant publications are likely well-represented. As PDTs continue to evolve, future research should aim for more balanced representation across all scientific domains to ensure not only efficacy but also usability, equity, and real-world impact.
